# The burden of motor neuron diseases in Asia, 1990–2021: temporal patterns and age-period-cohort analyses

**DOI:** 10.3389/fneur.2025.1640190

**Published:** 2025-09-08

**Authors:** Tianyu Shi, Zhiheng Qian, Tao Liu, Peng Zhang

**Affiliations:** Department of Orthopedics, The Second Affiliated Hospital of Soochow University, Suzhou, Jiangsu, China

**Keywords:** motor neuron disease, Global Burden of Disease, amyotrophic lateralsclerosis, epidemiologic studies, Asia

## Abstract

**Background:**

This study aims to characterize the temporal trends of motor neuron diseases (MNDs) burden in Asia from 1990 to 2021, estimate their age, period and cohort effects, and forecast the disease burden for the next 15 years.

**Methods:**

Data were obtained from the Global Burden of Disease study 2021. The average annual percentage change (AAPC) of MNDs incidence, prevalence, mortality and disability-adjusted life years (DALYs) in Asia from 1990 to 2021 were estimated by joinpoint regression analysis. Age, period and cohort effects on incidence and mortality of MNDs were also evaluated by the age-period-cohort analysis. The autoregressive integrated moving average model was used to forecast age-standardized incidence, prevalence and mortality rates of Asian MNDs in 2022–2036.

**Results:**

From 1990 to 2021, the age-standardized mortality and prevalence rates of Asian MNDs increased from 0.140 to 0.173/100,000, and from 2.134 to 2.187/100,000, respectively. While the age-standardized incidence rate in Asia decreased from 0.561/100,000 in 1990 to 0.478/100,000 in 2021. The AAPC in age-standardized incidence, prevalence, mortality and DALYs rates of MNDs in Asia were −0.508, 0.087, 0.649 and −0.045, respectively. The age-period-cohort analysis showed that, for mortality and incidence, the burden of MNDs increased with age. The period effect exhibited an initial decline followed by a subsequent resurgence. The cohort effect increased in the early birth cohort while declined in the recent birth cohort. By 2036, the prevalence and mortality of MNDs are projected to rise to 2.241/100,000 and 0.177/100,000, respectively, while incidence is projected to decline to 0.468/100,000.

**Conclusions:**

The age-standardized incidence rate of MNDs in Asia was reduced yet their age-standardized rates of prevalence and mortality increased. This trend is projected to persist into the foreseeable future. Given substantial population base and increasingly severe aging demographics in Asia population, a holistic strategy is required to ease the burden of MNDs in Asia and get better health results for Asian patients with MNDs.

## 1 Introduction

Motor neuron diseases (MNDs), a group of neurodegenerative diseases, feature the degeneration of upper and lower motor neurons (1), including amyotrophic lateralsclerosis (ALS), spinal muscular atrophy, primary lateral sclerosis, pseudobulbar palsy, progressive muscular atrophy and hereditary spastic paraplegia (2). As the commonest entity of MNDs, ALS leads to extensive paralysis and generally die of respiratory failure within a 2-year diagnosis of 50% patients (3, 4). With unfavorable long-range prognostic outlook as well, other MNDs also impose a heavy socio-economic burden on sufferers and healthcare institutions (5, 6).

Over the past three decades, epidemiological studies about MNDs were relied on Europe and the United States, and relevant systems of epidemiological surveillance are unavailable in Asia and other regions (7, 8). Mounting evidence demonstrated that MNDs are differently risky across continents and ethnicities (9). Asian population takes up over 50% of the global population (10). The incidence and prevalence of MNDs in Asia are much lower than those in North America and Europe, but MNDs in Asia constitute a non-negligible burden due to large population (11). As progressive neurodegenerative disorder influenced by aging, ALS exhibits high incidence in the elderly (12–14). Moreover, geriatric patients tend to develop more severe clinical phenotype and display worse prognosis compared to young patients (15, 16). Life expectancy in countries with huge demographic impacts experiences a fast increase. Therefore, studies predict that the burden of MNDs will shift from developed countries to developing countries in the coming decades (9, 17). To address this scenario, Asia, which is a cluster of developing countries, needs sound epidemiological assessments to guide the implementation of health interventions.

As a frequently used demographic, sociological and epidemiological model, the age-period-cohort model is used for evaluating three different time-related variations including age, period and cohort effects on disease burden (18). Cohort effects delineate variations in disease risk for individuals born in the same period, mainly associated with prolonged environmental exposures. Period effects reflect external elements that influence an entire population during particular period, which can be explained by changes in socioeconomic factors (19). However, to date there is no study employed the age-period-cohort model to analyze the burden of MNDs in Asia.

Although existing global epidemiologic studies of MNDs, the burden of MNDs mainly in Asia was not deeply analyzed. For filling the gap, this study aims to estimate the burden of MNDs in Asia, including incidence, prevalence, mortality, disability-adjusted life years (DALYs), years of life lost (YLLs) and years lived with disability (YLDs) from 1990 to 2021 based on the Global Burden of Disease Study (GBD) 2021. Furthermore, the effects of age, period and cohort on the burden of Asian MNDs were evaluated, and disease burden throughout 2022 to 2036 was predicted.

## 2 Materials and methods

### 2.1 Data source

At present, disease modeling meta-regression, version 2.1 (DisMod-MR 2.1) is mainly used by the GBD database for estimation. DisMod-MR 2.1 is a Bayesian disease modeling meta-regression tool that generates internally consistent estimates of incidence, prevalence and mortality in light of relevant data. This study is based on the latest GBD 2021 database (https://ghdx.healthdata.org/gbd-2021), which provides detailed records of the prevalence, incidence, death and DALY rates of over 300 diseases and injuries by sex and age across 204 nations and regions (20). Prevalence, incidence, death, YLDs, YLLs and DALYs rates, as well as their 95% uncertainty intervals (UIs) for Asian and global MNDs from 1990 to 2021 were extracted and stratified by sex and age.

### 2.2 Socio-demographic index

Socio-demographic index (SDI) as an aggregative indicator measures the level of socio-demographic development in a nation or region. The index makes it possible to compare different nations and regions regarding the association of socio-economic development with health burden. SDI is indicated on a scale from 0 to 1, where 0 represents the lowest per capita income and educational level, and the highest total birth rate across GBD regions, whereas 1 means the opposite (21).

### 2.3 DALYs

Computed by the summation of YLLs and YLDs, DALYs refer to the total loss of health caused by morbidity and mortality in a population. It is the most extensively used measure of health loss in GBD studies and provides policymakers with precious insights into the influence of different diseases, physical conditions or interventions on the health of the total population (21).

### 2.4 Joinpoint regression analysis

The secular trend with obvious changes in MNDs was examined using joinpoint regression analysis. Joinpoint software was employed for calculating annual percent change (APC) and average annual percent change (AAPC) as well as a 95% confidence interval (CI) for the incidence, prevalence, mortality and DALYs rates of Asian and global MNDs from 1990 to 2021. Best-fitting models were chosen for comparison, and the tendencies in disease burden were evaluated. It indicates an upward trend if the AAPC estimate has a 95% CI of above 0, a downward trend if the AAPC estimate has a 95% CI of below 0, and a stable trend if the AAPC estimate has a 95% CI equal to 0 (22).

### 2.5 Age-period-cohort analysis

An age-period-cohort model was utilized for developing the independent effect estimates of period, age and birth cohort on the incidence and mortality of MNDs (23). The results of the model demonstrated longitudinal age curves and period/cohort rate ratios (RR). The longitudinal age curves illustrated the fitted longitudinal age-specific rates within the reference cohort adjusted for periodic deviations, while the cohort/period RR quantified the cohort/period relative risk compared to the referent group. The data series was classified into consecutive 5-year intervals throughout 1992–2021. Data from 1990 to 1991 were not analyzed because they did not span a 5-year interval. Subjects were also divided into 5-year age groups ranging from 0–4 to 90–94 years old, and those aged above 94 were excluded from the age-period-cohort analysis. In addition, 24 cohorts were summarized and covered subjects born from 1902–1906 to 2017–2021. The mean level of age, period and cohort was selected as the reference group (24). The estimated parameters were obtained from the age-period-cohort Web Tool developed by the US National Cancer Institute [https://analysistools.cancer.gov/apc/ (25)].

### 2.6 Model prediction

Age-standardized rates of incidence, prevalence and mortality (ASIR, ASPR and ASMR, respectively) from 2022 to 2036 were predicted using an autoregressive integrated moving average (ARIMA) model (26). The ARIMA model makes a combination of autoregressive, integration and moving average components for effectively capturing cyclical patterns and trends in time series data and predicting possible changes on the basis of current data. In the ARIMA (p, d, q) model, “p,” “d,” and “q” denote the number of autoregressive terms, differences and moving average terms, respectively. In this research, the auto.arima() function of R software was applied to select the best ARIMA(p, d, q) model for projecting disease incidence and mortality trends from 2022 to 2036.

### 2.7 Statistical analysis

The data were statistically analyzed and visualized by use of Joinpoint (version 5.1.0.0) and R software (version 4.3.1). It was considered that all the analyses showed significance in the case of *P*-value < 0.05.

## 3 Results

### 3.1 Descriptive analysis

In Asia, 103,790 (95% UI: 84,254, 125,082) cases of MNDs were found in 2021, including newly diagnosed 22,712 (95% UI: 19,411, 26,490). MNDs resulted in 8,553 (95% UI: 7,010, 10,160) deaths. ASPR, ASIR, ASMR, as well as age-standardized rates of DALYs (ASDR), YLDs, and YLLs in 2021 were 2.187 cases (95% UI: 1.778, 2.636), 0.478 new cases (95% UI: 0.412, 0.555), 0.173 deaths (95% UI: 0.142, 0.205), 5.118 DALYs (95% UI: 4.056, 6.036), 0.465 YLDs (95% UI: 0.313,0.650) and 4.652 YLLs (95% UI: 3.596, 5.583) per 100,000 (Table 1). Both males and females' all-age cases and age-standardized rates are also demonstrated in Table 1. The disease burden of males was higher than that of females.

**Table 1:** All-ages cases and age-standardized DALYs, deaths, prevalence, incidence, YLDs and YLLs rates in 1990 and 2021 for MNDs in Asia.

**Measure**	**All-ages cases**			**Age-standardized rates per 100,000 people**		
	***n*** **(95% UI)**			***n*** **(95% UI)**		
	**Male**	**Female**	**Both**	**Male**	**Female**	**Both**
**1990**						
DALYs	78,595 (38,092, 101,007)	64,435 (58,465, 72,141)	143,030 (102,840, 169,392)	5.683 (3.122, 7.068)	4.707 (4.291, 5.200)	5.185 (3.891, 6.049)
Deaths	1,767 (986, 2,195)	1,401 (1,270, 1,535)	3,168 (2,410, 3,665)	0.160 (0.102, 0.191)	0.122 (0.111, 0.133)	0.140 (0.112, 0.159)
Prevalence	34,313 (27,552, 41,759)	30,587 (24,448, 37,686)	64,900 (52,069, 79,427)	2.209 (1.809, 2.641)	2.057 (1.672, 2.489)	2.134 (1.739, 2.562)
Incidence	8,411 (7,256, 9,807)	7,051 (6,061, 8,200)	15,463 (13,357, 18,059)	0.601 (0.519, 0.696)	0.521 (0.447, 0.608)	0.561 (0.482, 0.652)
YLDs	7,299 (4,839, 10,230)	6,507 (4,276, 9,302)	13,805 (91,34, 19,496)	0.470 (0.316, 0.655)	0.438 (0.293, 0.616)	0.454 (0.303, 0.634)
YLLs	71,296 (30,164, 94,181)	57,928 (52,108, 65,343)	129,224 (89,298, 155,197)	5.213 (2.613, 6.633)	4.270 (3.854, 4.722)	4.731 (3.468, 5.560)
**2021**						
DALYs	146,538 (93,612, 191,221)	106,233 (94,005, 119,655)	252,771 (201,243, 301,064)	6.049 (3.901, 7.772)	4.234 (3.756, 4.741)	5.118 (4.056, 6.036)
Deaths	4,899 (3,391, 6,187)	3,654 (3,153, 4,206)	8,553 (7,010, 10,160)	0.210 (0.150, 0.259)	0.141 (0.122, 0.162)	0.173 (0.142, 0.205)
Prevalence	54,748 (44,440, 65,377)	49,042 (39,739, 59,409)	103,790 (84,254, 125,082)	2.300 (1.870, 2.740)	2.078 (1.689, 2.525)	2.187 (1.778, 2.636)
Incidence	12,395 (10,659, 14,464)	10,317 (8,776, 12,046)	22,712 (19,411, 26,490)	0.527 (0.458, 0.607)	0.432 (0.367, 0.503)	0.478 (0.412, 0.555)
YLDs	11,642 (7,833, 16,233)	10,430 (7,031, 14,480)	22,072 (14,839, 30,704)	0.489 (0.330, 0.682)	0.442 (0.296, 0.624)	0.465 (0.313, 0.650)
YLLs	134,896 (82,057, 178,351)	958,03 (84,244, 108,702)	230,699 (17,9179, 276,855)	5.560 (3.406, 7.234)	3.792 (3.350, 4.279)	4.652 (3.596, 5.583)

MNDs, motor neuron diseases; DALYs, disability-adjusted life years; YLDs, years lived with disability; YLLs, years of life lost; UI, uncertainty interval.

**Figure 1:**
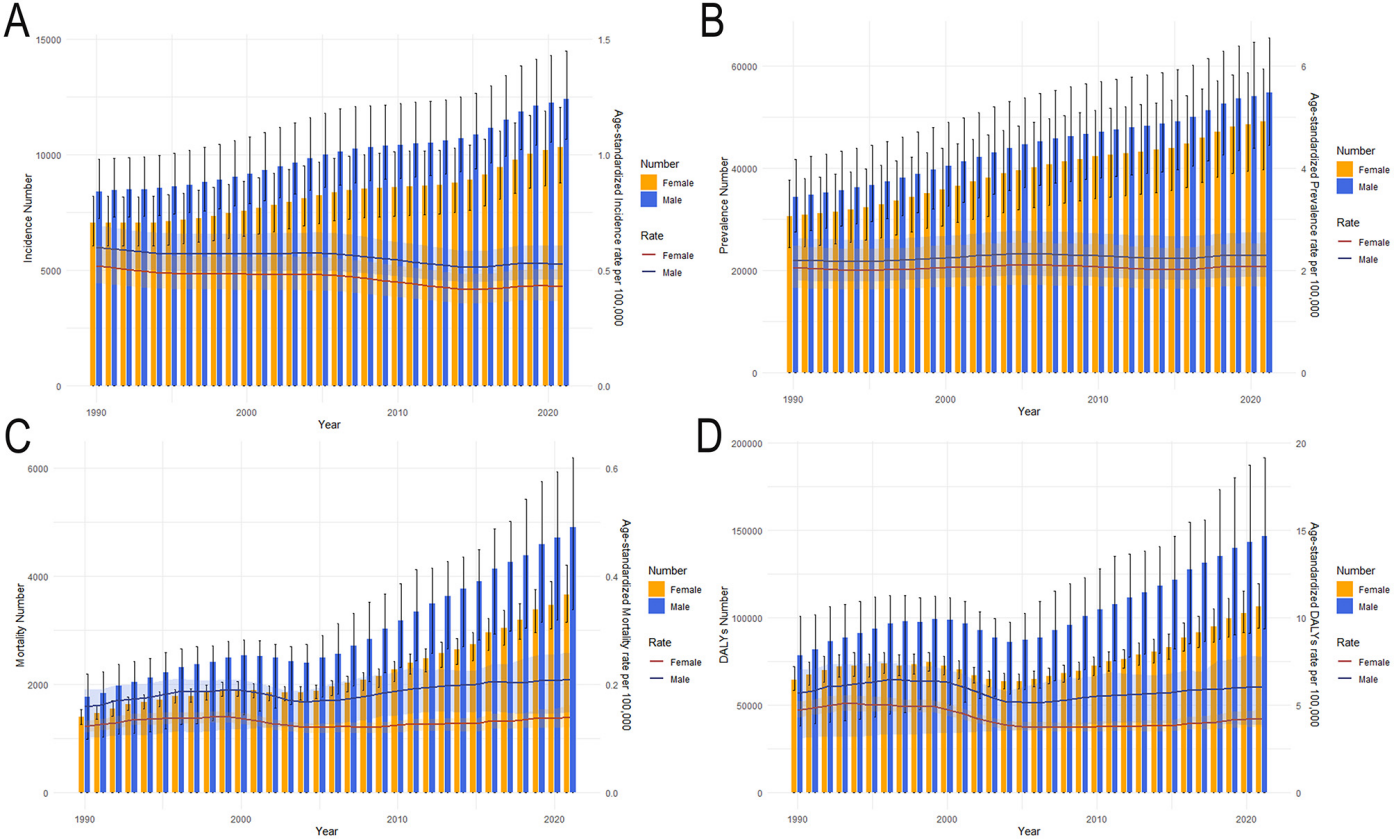
Trends in the all-age cases and age-standardized incidence, prevalence, mortality and DALYs rates of Asian MNDs by sex from 1990 to 2021. **(A)** Incidence number and rate. **(B)** Prevalence number and rate. **(C)** Mortality number and rate. **(D)** DALYs number and rate. Shaded regions represent the 95% UI. MNDs, motor neuron diseases; DALYs, disability-adjusted life years; 95% UI, 95% uncertainty interval.

The tendencies in the sex-specific all-age number and ASIR, ASPR, ASMR, ASDR, as well as age-standardized YLDs and YLLs rates of Asian MNDs from 1990 to 2021 are depicted in Figure 1 and Supplementary Figure 1. Between 2019 and 2021, ASMR and ASPR in the total population exhibited an increase and changed from 0.140 to 0.173 per 100,000 and from 2.134 to 2.187 per 100,000, respectively. The age-standardized YLDs rate in 2021 also increased compared to that in 2019, while ASIR in the total population demonstrated a decline during the same period and changed from 0.561 to 0.478 per 100,000. Although ASDR and age-standardized YLLs rates in the total population decreased, the ASDR and the age-standardized YLLs rates of males in 2021 increased compared to those in 2019. The age-standardized rates of these indicators exhibited varying trends, but their all-age numbers in both sexes all showed a steady increase from 1990 to 2021, which may imply that the growing burden of MNDs in Asia is primarily influenced by demographic shifts.

**Figure 2:**
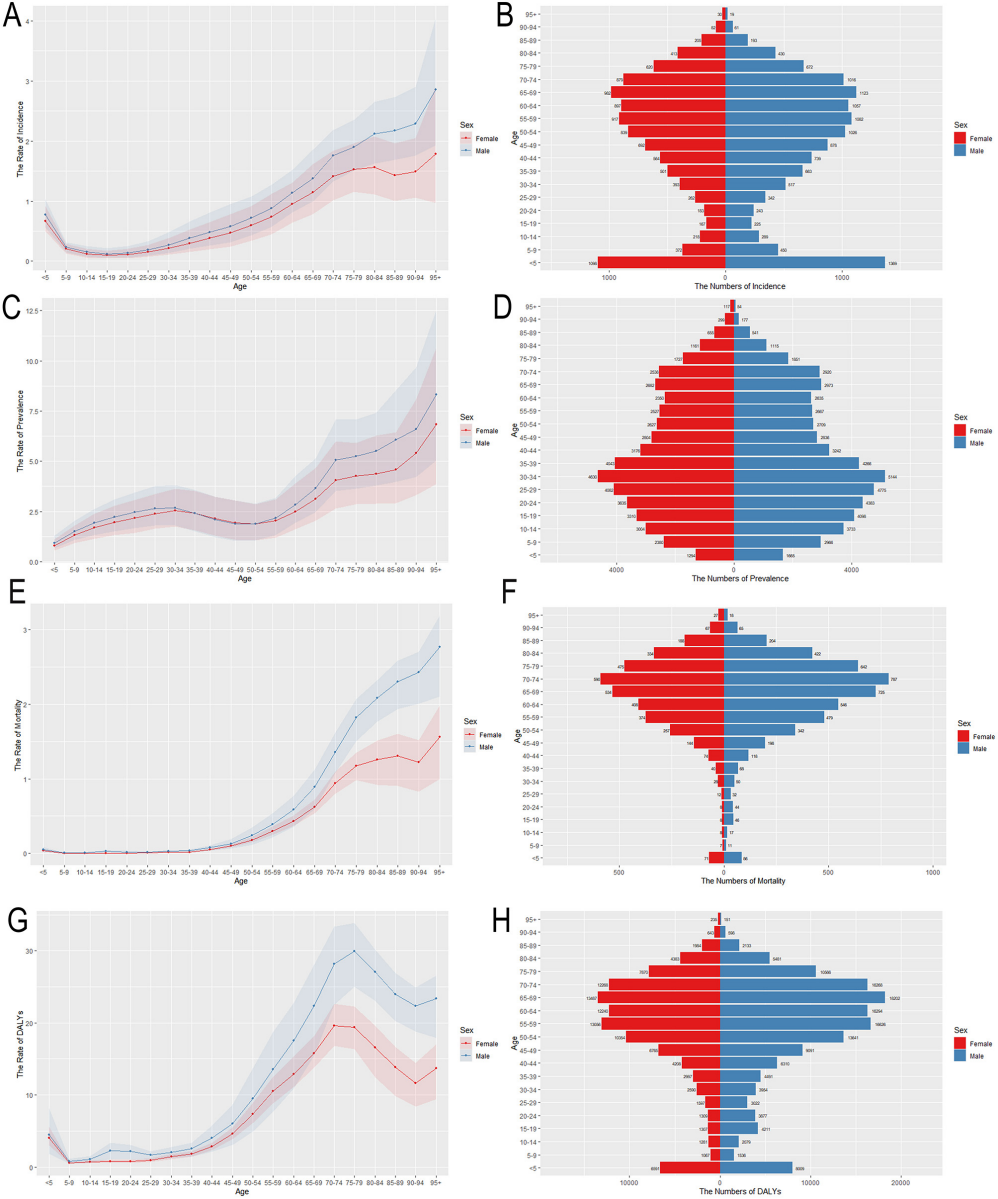
Age-specific numbers and age-standardized incidence, prevalence, mortality and DALYs rates of MNDs in Asia, 2021. **(A)** Age-standardized incidence rate. **(B)** Age-specific incidence number. **(C)** Age-standardized prevalence rate. **(D)** Age-specific prevalence number. **(E)** Age-standardized mortality rate. **(F)** Age-specific mortality number. **(G)** Age-standardized DALYs rate. **(H)** Age-specific DALYs number. Shaded regions represent the 95% UI. MNDs, motor neuron diseases; DALYs, disability-adjusted life years; 95% UI, 95% uncertainty interval.

ASIR, ASPR, ASMR, ASDR, as well as age-standardized YLDs and YLLs rates of MNDs for various age groups in 2021 are also presented in Figure 2 and Supplementary Figure 2. The burden trends of MNDs in Asia demonstrated comparable patterns between sexes. ASMR escalated progressively with advancing age and was characterized by an accelerated trajectory in populations aged over 50. ASPR and age-standardized YLDs rates demonstrated similar trends by sex and age. ASIR exhibited an initial peak with a small scale in the under-five demographic, followed by a progressive decline to a nadir during adolescence. A progressive increase with advancing age subsequently commenced in the post-pubertal stage. In terms of DALYs and YLLs, their age-standardized rates exhibited a minor peak in the under-five demographic, followed by a decline. After adolescence, ASDR and age-standardized YLLs rates increased with age and declined slightly after peaking around the age of 75. Overall, males exhibited a higher disease burden across all metrics compared to females, with this sex disparity progressively widening after the age of 50.

### 3.2 Temporal patterns of MNDs burden in Asia

**Figure 3:**
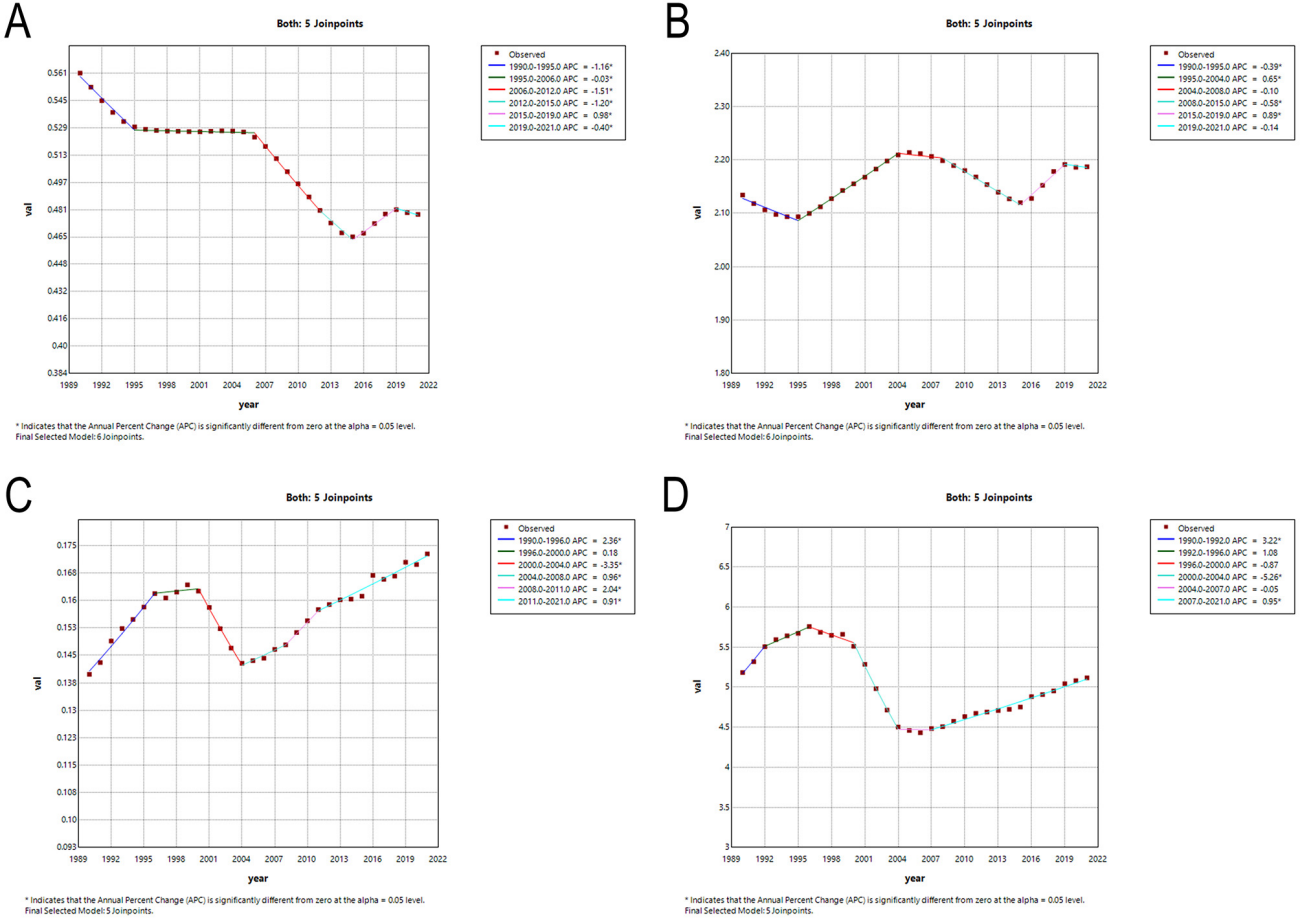
Temporal trends in MNDs burden in Asia between 1990 and 2021. **(A)** The age-standardized incidence rate. **(B)** The age-standardized prevalence rate. **(C)** The age-standardized mortality rate. **(D)** The age-standardized DALYs rate. MNDs, motor neuron diseases; DALYs, disability-adjusted life years; APC, annual percent change.

The segmental tendencies of the age-standardized rates of Asian MNDs from 1990 to 2021 were divided using joinpoint analysis (Figure 3 and Supplementary Table 1). The AAPCs in the incidence, prevalence, mortality and DALYs rates of MNDs from 1990 to 2021 were −0.508 (95% CI: −0.547, −0.470, *P* < 0.05), 0.087 (95% CI: 0.051, 0.123, *P* < 0.05), 0.649 (95% CI: 0.370, 0.929, *P* < 0.05), and −0.045 (95% CI: −0.348, 0.260, *P* > 0.05), respectively (Supplementary Table 1). The AAPCs for the remaining indicators except DALYs demonstrated statistical significance. ASIR demonstrated a notable decline from 1990 to 2015 and then a transient rise from 2015 to 2019 before resuming a sustained downward trajectory thereafter. ASPR demonstrated a dramatic decrease from 1990 to 1995, and then a remarkable increase from 2004 to 2008. After temporary stabilization, ASPR exhibited a significant decline followed by a marked increase. ASMR exhibited a significant increase from 1990 to 1996, and then remained steady from 1996 to 2000. ASMR showed a marked decrease from 2000 to 2004 and then tended to significantly increase from 2004 to 2021. ASDR exhibited a similar temporal pattern and featured a significant increase, a marked decline and a resurgence of remarkable upward trends.

### 3.3 Burden of MNDs by country and region

**Figure 4:**
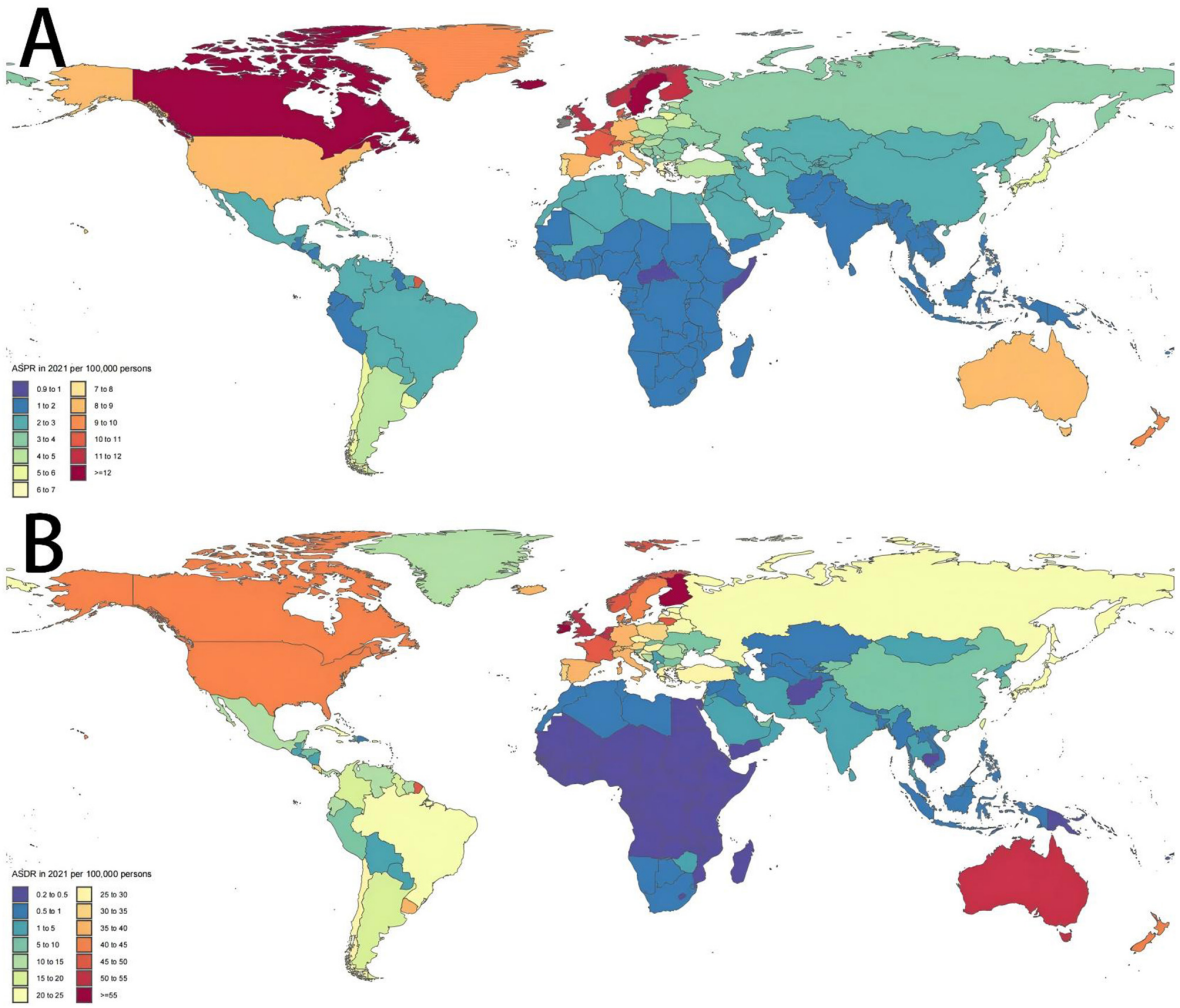
Age-standardized prevalence and DALYs rates in 2021 of MNDs in 204 countries or territories. **(A)** ASPR in 2021 of MNDs in 204 countries or territories. **(B)** ASDR in 2021 of MNDs in 204 countries or territories. MNDs, motor neuron diseases; DALYs, disability-adjusted life years; ASPR, age-standardized prevalence rate; ASDR, age-standardized DALYs rate.

**Figure 5:**
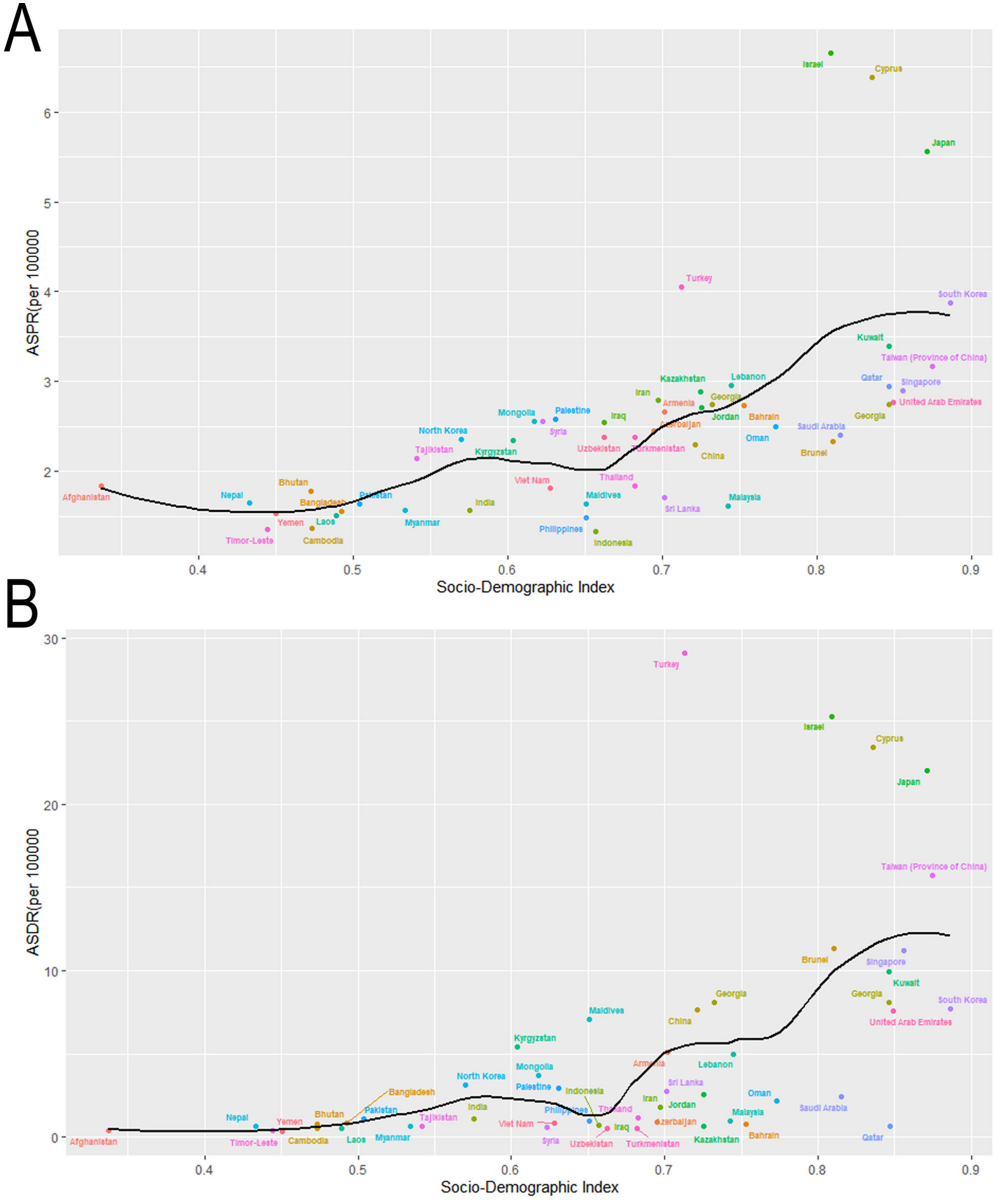
Age-standardized rates of prevalence and DALYs due to MNDs for Asian countries and territories by Socio-demographic Index in 2021. **(A)** ASPR due to MNDs for Asian countries and territories by Socio-demographic Index in 2021. **(B)** ASDR due to MNDs for Asian countries and territories by Socio-demographic Index in 2021. MNDs, motor neuron diseases; DALYs, disability-adjusted life years; ASPR, age-standardized prevalence rate; ASDR, age-standardized DALYs rate.

Across the globe, regions with higher SDI values, including Australia, New Zealand, Western Europe and North America, exhibited significantly elevated ASPR of MNDs in 2021 compared to those with lower SDI levels, like parts of Asia and Africa. Within Asia, a parallel trend was observed: Subregions with higher SDI like East Asia demonstrated a greater burden of MNDs relative to subregions with lower SDI like South Asia and Southeast Asia (Figure 4A). This disparity was similarly reflected in the ASDR of MNDs in 2021 (Figure 4B). In Asia, the detected national ASPR and ASDR regarding SDI vs. the anticipated levels for every country on the basis of SDI are displayed (Figure 5). The results broadly indicated that the burden estimates of MNDs were positively correlated with the SDI levels of each Asian country during observation.

### 3.4 Effects of age, period and cohort on the incidence and mortality of MNDs

**Figure 6:**
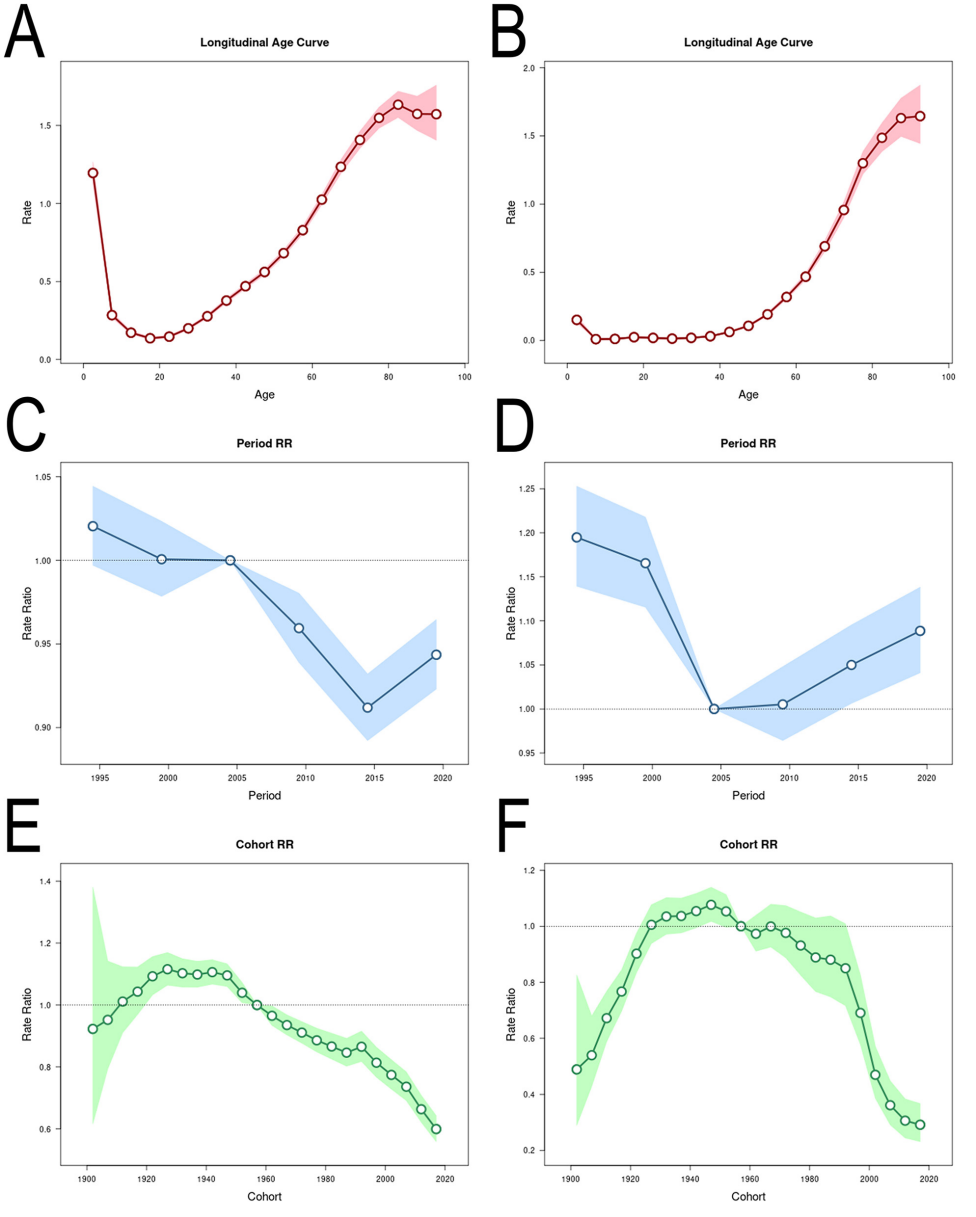
Effects of age, period and cohort on the incidence and mortality of MNDs. Longitudinal age curves of MNDs incidence **(A)** and mortality **(B)** in Asia. Period rate ratios of MNDs incidence **(C)** and mortality **(D)** in Asia. Cohort rate ratios of MNDs incidence **(E)** and mortality **(F)** in Asia. Dotted lines represtented the level of the reference one. Shaded regions represent 95% CI. MNDs, motor neuron diseases; RR, rate ratio; 95% CI, 95% confidence intervals.

The effects of age, period and cohort on the incidence and mortality rate of MNDs in Asia were estimated (Figure 6). The estimation results were statistically significant (Supplementary Table 2). For mortality, the burden of MNDs increased with age. The period RR exhibited a decline until around 2005, followed by a subsequent resurgence. The cohort RR increased in pre-1947 cohorts and then remained stable temporarily. The cohort RR sharply decreased in the cohorts born after 1992.

For incidence, the burden of MNDs in the age group of 0–4 exhibited a peak and then declined to a relatively low level. Subsequently, the incidence of MNDs increased with age since the age of 20. The period RR exhibited a decline until around 2015, followed by a subsequent resurgence. The cohort RR increased in pre-1927 cohorts and then remained stable temporarily. In the cohorts born after 1947, the cohort RR demonstrated a continuous decrease.

### 3.5 Prediction of ASIR, ASPR, and ASMR of MNDs in Asia

**Figure 7:**
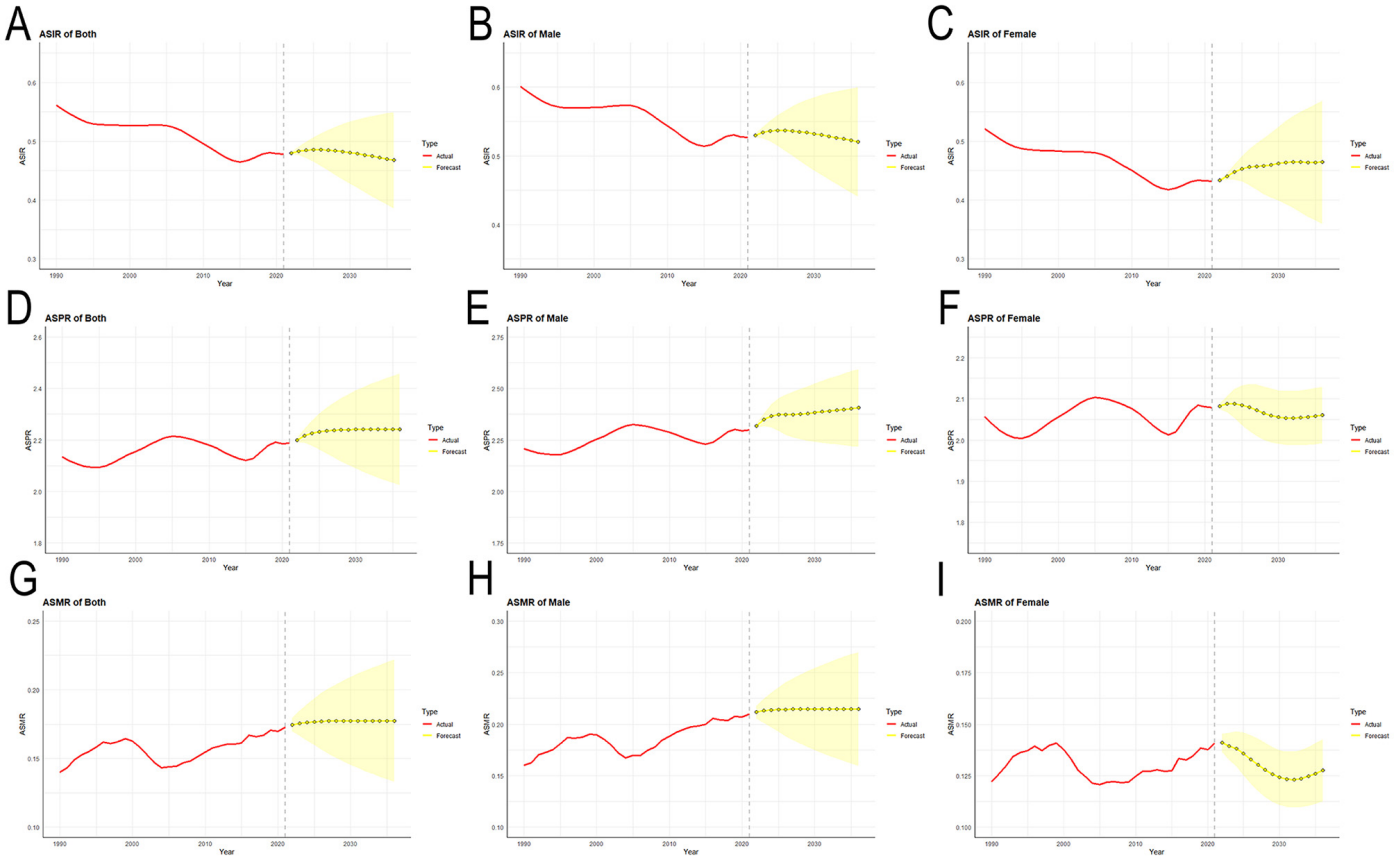
Predicted trends of MNDs incidence, prevalence and mortality age-standardized rates in Asia over the next 15 years (2022–2036). **(A)** ASIR for both sexes. **(B)** ASIR for males. **(C)** ASIR for females. **(D)** ASPR for both sexes. **(E)** ASPR for males. **(F)** ASPR for females. **(G)** ASMR for both sexes. **(H)** ASMR for males. **(I)** ASMR for females. Red lines represent the true trend of MNDs incidence, prevalence and mortality rates during 1990–2021; yellow dot lines and shaded regions represent the predicted trend and its 95% CI. MNDs, motor neuron diseases; ASIR, age-standardized incidence rate; ASPR, age-standardized prevalence rate; ASMR, age-standardized mortality rate; 95% CI, 95% confidence intervals.

The auto.arima() function was applied to the data of MNDs from Asia between 1990 and 2021. Trends for the coming 15 years were projected (Figure 7). The best models were selected for ASIR, ASPR and ASMR. The results of the ARIMA model show that the ASPR and ASMR of MNDs for the total Asian population will increase in the coming 15 years, while ASIR will decline. From 2021 to 2036, it is expected that the ASIR of MNDs in all Asian people will decrease from 0.478 to 0.468 per 100,000. In addition, the ASIR of MNDs will decrease from 0.527 to 0.520 per 100,000 for males, but increase from 0.432 to 0.464 per 100,000 for females. It is projected that the ASPR of MNDs for all Asian people will increase from 2.187 to 2.241 per 100,000. Additionally, the ASPR of MNDs for males will increase from 2.300 to 2.406 per 100,000, while that for females will decline from 2.078 to 2.060 per 100,000. It is predicted that the ASMR of MNDs for all Asian people will increase from 0.173 to 0.177 per 100,000. Moreover, the ASMR of MNDs for males will increase from 0.210 to 0.215 per 100,000, while that for females will decline from 0.141 to 0.128 per 100,000.

## 4 Discussion

Asia is confronting intensifying population aging and holds the highest absolute number of elderly individuals worldwide (27). Given the higher incidence rates and poorer prognoses of MNDs among aging populations (12–14), the demographic transition may exacerbate the burden of MNDs in Asia. However, epidemiological research focusing on the MNDs burden in Asia is lacking. In this study, the trends in the burden of MNDs in Asia from 1990 to 2021 were examined based on the data from GBD 2021. This is the first study to analyze the epidemiological trends of MNDs in Asia using joinpoint analysis combined with age-period-cohort and ARIMA models.

From 2019 to 2021, ASPR and ASMR of Asian MNDs increased, while ASDR and ASIR decreased. Although the age-standardized rates of these indicators exhibited varying trends, their all-age numbers in both sexes displayed a significant increase, which indicated the growing burden of MNDs in Asia. Population growth and aging exacerbation may have a more substantial influence on the escalation of disease burden (9, 28). Based on the fact that many developing countries in Asia have a great demographic impact and quickly increasing life expectancy (9), projection studies suggested an impending epidemiological shift, with the MNDs burden transitioning from Western countries to Asian nations (17).

From 1990 to 2021, ASIR of MNDs in Asia generally exhibited a downward trajectory. This epidemiological trajectory diverges from patterns observed in Western countries, where historical data demonstrated a upward progression in MNDs incidence (11). The fast modernization of Asian countries changing environment and lifestyle may influence incidence by removing an unknown exposure to MNDs (29, 30). Furthermore, the application of updated diagnostic criteria and accessibility to medical resources influenced by regional economic level may partly introduce geographical differences in the assessment of incidence (7). The growth in ASPR and the decline in ASDR could have been influenced by the development of therapeutic methods like the widespread utilization of non-invasive ventilators in ALS or the application of new-type drugs such as Nusinersen, Risdiplam, and Onasemnogene abeparvovec used for treating spinal muscular atrophy (31, 32). Moreover, males exhibited a higher disease burden compared to females, which is consistent with previous reports (11, 33). Sex difference is involved in reactions to exogenous toxins, the discrepancies in exposure to environmental hazards, the structure of the nervous system and the ability to damage correction (33).

Consistent with prior studies (11), Asia exhibited lower prevalence and DALYs of MNDs compared to Europe and America, which indicated that geography exerted a significant impact on some clinical characteristics (9). Several potential factors may contribute to the observed regional disparities. First, Asian people exhibited longer survival when the median age of onset of ALS was younger, and age was the strongest influence factor of prognosis in ALS (9, 12, 15, 16). Second, the proportions of some severe ALS subtypes with worse prognosis in Asia are smaller than those in Europe, which may influence disease progression (4, 9, 34). Third, tracheostomy positive pressure ventilation identified as a useful was used by over 20% of patients with ALS in Taiwan and Japan, while the utility in western countries was below 10% (35, 36). Furthermore, the low incidence of chromosome 9 open reading frame 72 repeat expansion associated with MNDs in Asian populations may be responsible for these differences to some extent (9, 37, 38). We also found that Asian regions with higher SDI levels tend to exhibit higher prevalence and DALYs. This may be because patients in high-income regions have enhanced accessibility to healthcare interventions and benefit from superior therapeutic quality, which contributes to prolonged survival time (11).

Age-period-cohort analysis showed the incidence and mortality of MNDs increased with aging. After age of 50–54, the age effect demonstrated roughly exponential risk trends. Previous studies exhibited that incidence of ALS increased with aging and geriatric patients tended to display poor prognosis with high mortality (9, 12, 15, 16). Incidence exhibited a slight decline after the age of 80, which may be caused by difficult diagnostic ascertainment in elderly patients. In general, it is tougher to identify ALS mimic syndromes in old people, and other deadly comorbidities are familiar. Moreover, old patients have a lower frequency of being transferred to a tertiary center because that frailty in old age is often thought to be the result of aging rather than pathology (11). The post-neonatal period sees another small incidence peak. The high rate of incidence in early childhood may result from the inclusion of MNDs instead of ALS occurring primarily in childhood, like hereditary spastic paraplegia and spinal muscular atrophy (39).

Period RRs on the incidence and mortality of Asian MNDs both demonstrated a comparable temporal pattern and exhibited an initial decline and then a subsequent rise in their trajectories. Since 1990, Asia has witnessed a constellation of transformative changes including enhanced economic openness, the westernization of lifestyle and living environments, the heightened awareness of preventing MNDs and the implementation of therapeutic interventions like Riluzole (40). These changes are likely to have collectively resulted in the detected decline in incidence and death rates. However, period RRs on the incidence and mortality resurged after 2016 and 2006, respectively, and were potentially mediated by shifts in exogenous environmental factors proximate to these temporal junctures. Future investigations are warranted to elucidate the drivers that underlie these epidemiological trend reversals.

The cohort RRs on incidence and mortality increased in pre-1927 cohorts. This may be attributed to the gradual introduction of the conceptual framework of MNDs in earlier medical practice coupled with the progressive dissemination of advanced diagnostic modalities during this phase. In the cohorts born after 1947, the cohort RRs on incidence and mortality decreased, which may be caused by the development of therapies, the westernization of lifestyle and economic improvement over the last half century (29, 30).

Following the ARIMA model, it is forecast that the prevalence and death rates of MNDs in the total Asian population will increase to 2.241 and 0.177 per 100,000 in 2036. However, among plenty of developing nations in Asia, the ability of patients to afford the comprehensive care of MNDs remains significantly constrained due to constraints in healthcare and financial resources (41, 42). Thus, a holistic strategy that contains the adjustment of regional medical resource allocation, the screening of MNDs among high-risk populations and access to medical care of high quality is required.

These data indicate that as population aging intensifies, the burden of MNDs on health services in Asia may increase in the future. In response to this situation, certain initiatives warrant consideration when devising adjustment strategies concerning healthcare planning and resource allocation. Early diagnosis of MNDs facilitates timely intervention and modification of disease prognosis (43). In Asia, the current diagnostic framework primarily relies on clinical manifestations of motor neuron involvement and electrophysiological evidence (44). Despite certain limitations, genetic testing remains a valuable supplementary tool for early clinical diagnosis and screening due to its rapid and accurate detection capabilities (43). Pharmacological interventions for MNDs remain under investigation (45, 46). The mainstream medication Riluzole provides modest survival benefits, while newer agents such as edaravone, sodium phenylbutyrate and taurursodiol are gaining approval in a growing number of countries (44). Consequently, palliative care assumes a prominent role in the management of MNDs. In the provision of palliative care, tracheostomy is implemented considerably more frequently in Asian countries compared to Western nations, whereas non-invasive ventilation demonstrates relatively lower utilization rates (11). Furthermore, social participation enhances psychological wellbeing in patients with MNDs and contributes substantively to improving their overall quality of life (43, 47). The implementation of these diverse measures necessitates robust healthcare policy support. Asian nations should undertake strategic reallocation of resources to facilitate the development of comprehensive diagnostic and therapeutic infrastructure, thereby addressing the mounting burden of MNDs.

This study suffers from the general limitations that are unavoidably present in the planning of GBD studies (48, 49). Firstly, information quality varies by country, and data may not be complete. Due to the lack of access to the raw dataset, bias could not be reduced by conducting further statistical analysis. Moreover, it is challenging to diagnose MNDs in clinical practice, and certain categories, particularly in older patients or ethnic minorities, are likely to be underdiagnosed. Lastly, the results at the population level were revealed by the age-period-cohort analysis and will be affected by ecological fallacy.

## 5 Conclusions

The age-standardized incidence rate of MNDs in Asia was reduced yet their age-standardized rates of prevalence and mortality increased. This trend is projected to persist into the foreseeable future. The current trajectory of MNDs burden poses a potential risk of exacerbating the strain on healthcare systems across Asian regions. Given substantial population base and increasingly severe aging demographics in Asia population, a holistic strategy is required to ease the burden of MNDs in Asia and get better health results for Asian patients with MNDs.

## Data Availability

Publicly available datasets were analyzed in this study. This data can be found here: https://ghdx.healthdata.org/gbd-2021.
