# Supplementary material for: The burden of motor neuron diseases in Asia, 1990–2021: temporal patterns and age-period-cohort analyses
Source: Front Neurol. 2025 Sep 8;16:1640190. doi: 10.3389/fneur.2025.1640190 (PMC12450676; doi:10.3389/fneur.2025.1640190)
Supplement: Supplementary file 1 [file Supplementary_file_1.docx]

**Supplementary Figures**


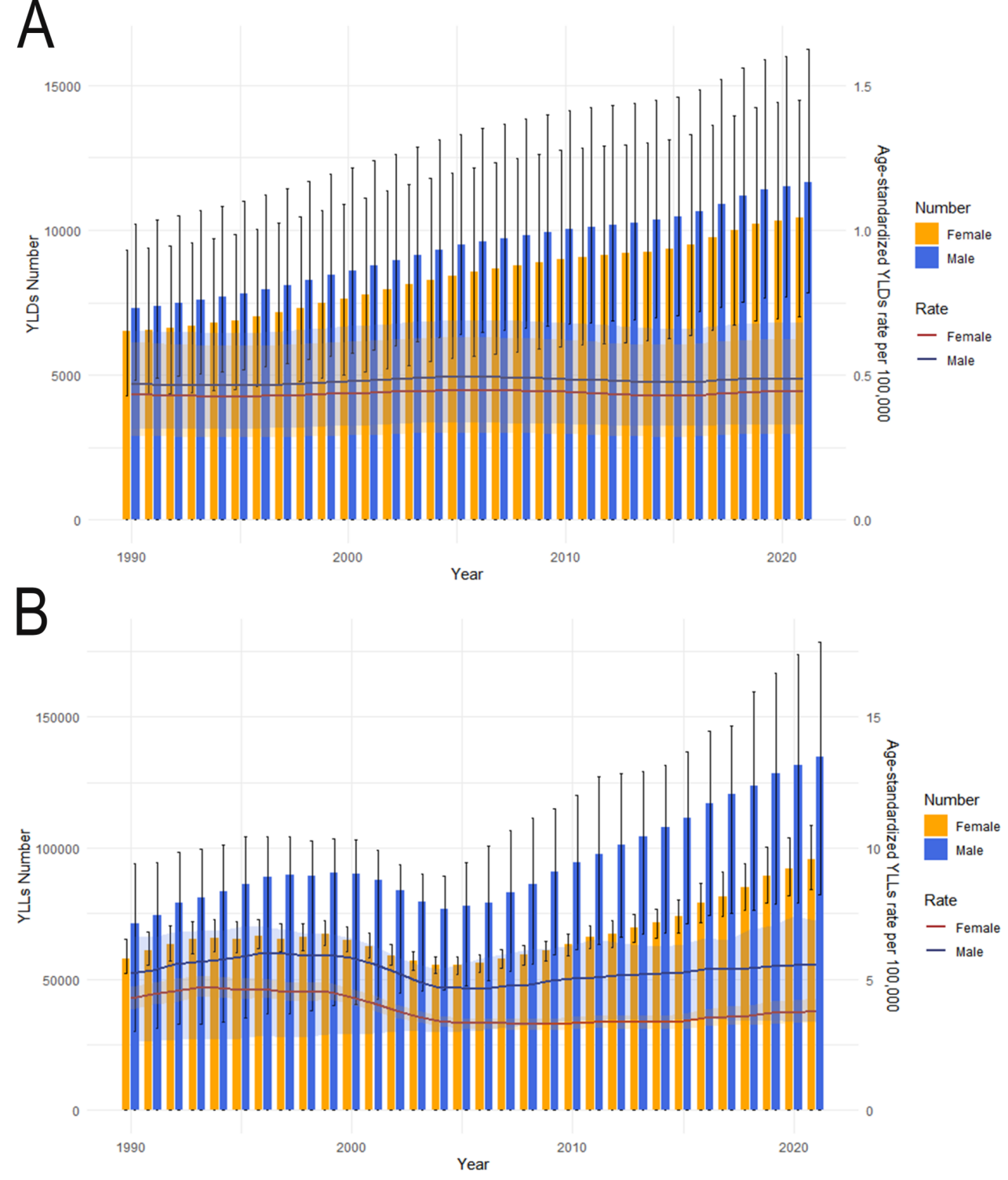


**Supplementary Figure 1.** Trends in the all-age cases and age-standardized YLDs and YLLs rates of MNDs by sex from 1990 to 2021. (A) YLDs number and rate. (B) YLLs number and rate. MNDs, motor neuron diseases; YLDs, years lived with disability; YLLs, years of life lost.


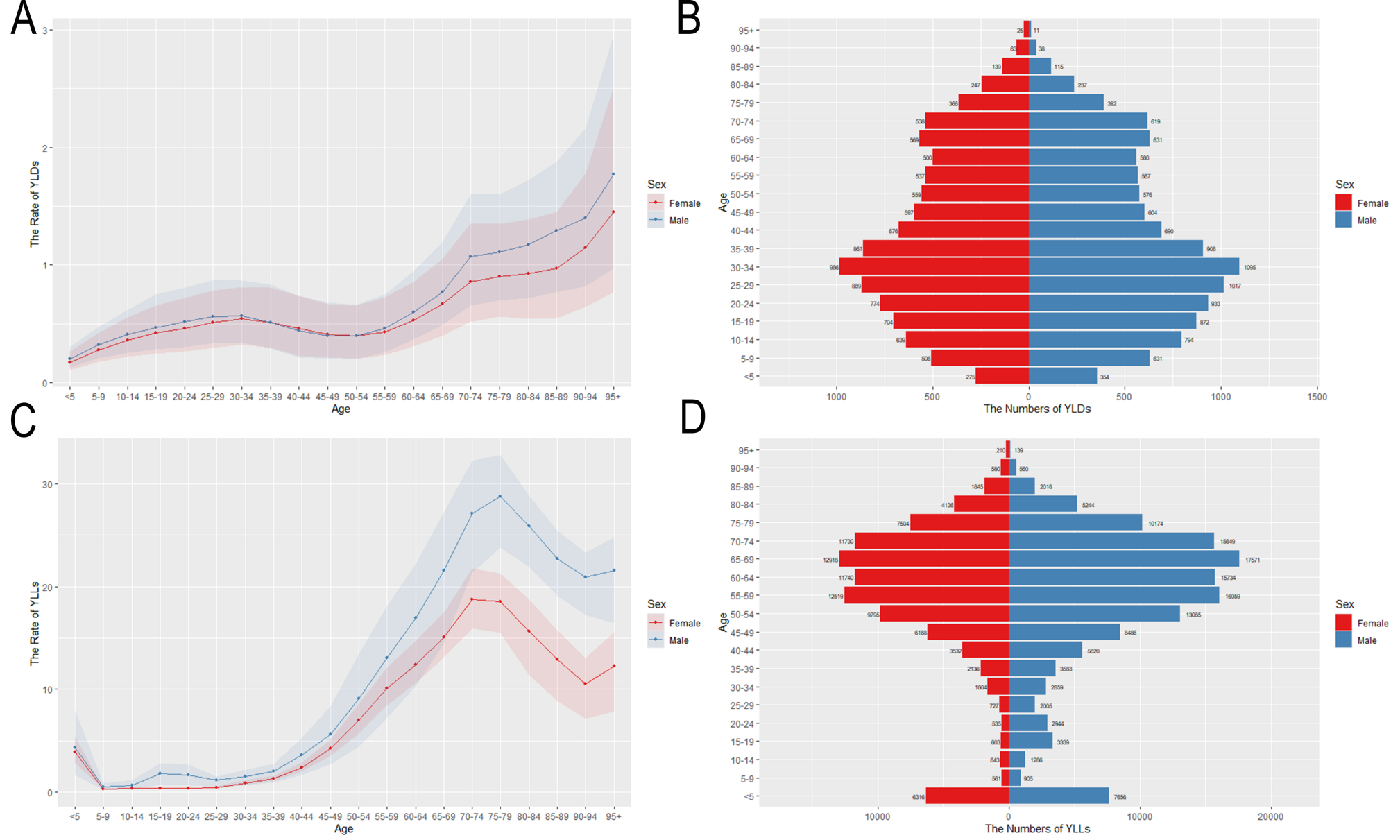


**Supplementary Figure 2.** Age-specific numbers and age-standardized YLDs and YLLs rates of MNDs in Asia, 2021. (A) Age-standardized YLDs rate. (B) Age-specific YLDs number. (C) Age-standardized YLLs rate. (D) Age-specific YLLs number. MNDs, motor neuron diseases; YLDs, years lived with disability; YLLs, years of life lost.
