# Supplementary material for: The burden of motor neuron diseases in Asia, 1990–2021: temporal patterns and age-period-cohort analyses
Source: Front Neurol. 2025 Sep 8;16:1640190. doi: 10.3389/fneur.2025.1640190 (PMC12450676; doi:10.3389/fneur.2025.1640190)
Supplement: Supplementary file 2 [file Table_1.docx]

| Gender | ASIR | | | ASPR | | | ASMR | | | ASDR | | |
| --- | --- | --- | --- | --- | --- | --- | --- | --- | --- | --- | --- | --- |
|  | Period | APC(95%CI) | AAPC(95%CI) | Period | APC(95%CI) | AAPC(95%CI) | Period | APC(95%CI) | AAPC(95%CI) | Period | APC(95%CI) | AAPC(95%CI) |
| Both | 1990-1995 | -1.158(-1.222,-1.093) | -0.508(-0.547,-0.470) | 1990-1995 | -0.394(-0.462,-0.326) | 0.087(0.051,0.123) | 1990-1996 | 2.359(1.942,2.779) | 0.649(0.370,0.929) | 1990-1992 | 3.219(0.749,5.750 ) | -0.045(-0.348,0.260) |
|  | 1995-2006 | -0.027(-0.050,-0.005) |  | 1995-2004 | 0.654(0.620,0.687) |  | 1996-2000 | 0.184(-0.948,1.328) |  | 1992-1996 | 1.079(-0.103,2.276) |  |
|  | 2006-2012 | -1.511(-1.575,-1.448) |  | 2004-2008 | -0.099(-0.251,0.053) |  | 2000-2004 | -3.346(-4.250,-2.434) |  | 1996-2000 | -0.871(-1.967,0.237) |  |
|  | 2012-2015 | -1.200(-1.478,-0.922) |  | 2008-2015 | -0.578(-0.630,-0.527) |  | 2004-2008 | 0.963(0.047,1.887) |  | 2000-2004 | -5.259(-6.136,-4.373) |  |
|  | 2015-2019 | 0.978(0.835,1.122) |  | 2015-2019 | 0.887(0.730,1.043) |  | 2008-2011 | 2.040(0.174,3.940) |  | 2004-2007 | -0.051(-1.674,1.599) |  |
|  | 2019-2021 | -0.404(-0.697,-0.110) |  | 2019-2021 | -0.139(-0.447,0.170) |  | 2011-2021 | 0.911(0.738,1.084) |  | 2007-2021 | 0.951(0.856,1.046) |  |
| Female | 1990-1994 | -1.509(-1.600,-1.418) | -0.601(-0.640,-0.562) | 1990-1995 | -0.528(-0.599,-0.456) | 0.042(0.006,0.079) | 1990-1993 | 3.401(2.010,4.811) | 0.469(0.199,0.739) | 1990-1993 | 2.543(1.355,3.745) | -0.329(-0.555,-0.102) |
|  | 1994-1997 | -0.315(-0.597,-0.031) |  | 1995-2004 | 0.578(0.542,0.613) |  | 1993-1999 | 0.772(0.256,1.291) |  | 1993-1999 | -0.476(-0.913,-0.038) |  |
|  | 1997-2006 | -0.123(-0.155,-0.092) |  | 2004-2009 | -0.132(-0.233,-0.031) |  | 1999-2004 | -3.022(-3.708,-2.330) |  | 1999-2004 | -4.937(-5.437,-4.433) |  |
|  | 2006-2015 | -1.592(-1.623,-1.561) |  | 2009-2015 | -0.661(-0.732,-0.589) |  | 2004-2008 | 0.239(-0.891,1.382) |  | 2004-2007 | -0.920(-2.469,0.654) |  |
|  | 2015-2019 | 1.159(1.011,1.306) |  | 2015-2019 | 0.944(0.781,1.107) |  | 2008-2015 | 0.829(0.381,1.280) |  | 2007-2015 | 0.377(0.134,0.621) |  |
|  | 2019-2021 | -0.358(-0.656,-0.059) |  | 2019-2021 | -0.171(-0.496,0.155) |  | 2015-2021 | 1.419(0.804,2.038) |  | 2015-2021 | 1.714(1.291,2.138) |  |
| Male | 1990-1995 | -1.026(-1.090,-0.962) | -0.419(-0.456,-0.382) | 1990-1995 | -0.279(-0.343,-0.216) | 0.135(0.096,0.173) | 1990-1996 | 2.545(2.096,2.996) | 0.891(0.588,1.194) | 1990-1996 | 2.099(1.763,2.435) | 0.190(-0.047,0.428) |
|  | 1995-2006 | 0.065(0.043,0.087) |  | 1995-2004 | 0.735(0.703,0.766) |  | 1996-2000 | 0.675(-0.524,1.888) |  | 1996-2000 | -0.518(-1.386,0.358) |  |
|  | 2006-2012 | -1.356(-1.418,-1.295) |  | 2004-2007 | -0.013(-0.296,0.271) |  | 2000-2004 | -3.287(-4.250,-2.314) |  | 2000-2004 | -5.068(-5.763,-4.367) |  |
|  | 2012-2015 | -0.995(-1.258,-0.731) |  | 2007-2015 | -0.510(-0.548,-0.472) |  | 2004-2007 | 1.293(-0.719,3.347) |  | 2004-2007 | 0.289(-1.127,1.726) |  |
|  | 2015-2019 | 0.892(0.757,1.028) |  | 2015-2019 | 0.840(0.695,0.986) |  | 2007-2011 | 2.793(1.728,3.868) |  | 2007-2010 | 1.989(0.514,3.486) |  |
|  | 2019-2021 | -0.464(-0.744,-0.184) |  | 2019-2021 | -0.118(-0.404,0.169) |  | 2011-2021 | 0.825(0.655,0.995) |  | 2010-2021 | 0.871(0.759,0.983) |  |

Supplementary Table 1. Joinpoint regression analysis: trends in age-standardized incidence, prevalence, mortality, DALYs rates (per 100,000 persons) among both sexes, males and females in Asia, 1990-2021

DALYs, disability-adjusted life years; ASIR, age-standardized incidence rate; ASPR, age-standardized prevalence rate; ASMR, age-standardized mortality rate; ASDR, age-standardized DALYs rate; APC, annual percent change; AAPC, average annual percent change; CI, confidence interval.
