# Supplementary material for: The burden of motor neuron diseases in Asia, 1990–2021: temporal patterns and age-period-cohort analyses
Source: Front Neurol. 2025 Sep 8;16:1640190. doi: 10.3389/fneur.2025.1640190 (PMC12450676; doi:10.3389/fneur.2025.1640190)
Supplement: Supplementary file 3 [file Table_2.docx]

| Group | Effect | |
| --- | --- | --- |
|  | Mortality | Incidence |
| Age |  |  |
| 0-4 | 0.1503 | 1.1954 |
| 5-9 | 0.0089 | 0.284 |
| 10-14 | 0.0106 | 0.1716 |
| 15-19 | 0.0234 | 0.136 |
| 20-24 | 0.0181 | 0.1464 |
| 25-29 | 0.0128 | 0.1995 |
| 30-34 | 0.0184 | 0.2769 |
| 35-39 | 0.0303 | 0.3778 |
| 40-44 | 0.0616 | 0.4698 |
| 45-49 | 0.1065 | 0.5609 |
| 50-54 | 0.191 | 0.6815 |
| 55-59 | 0.3183 | 0.8287 |
| 60-64 | 0.4673 | 1.0248 |
| 65-69 | 0.69 | 1.2352 |
| 70-74 | 0.9563 | 1.4071 |
| 75-79 | 1.2994 | 1.5481 |
| 80-84 | 1.4858 | 1.6331 |
| 85-89 | 1.6302 | 1.5737 |
| 90-94 | 1.6445 | 1.572 |
| Period |  |  |
| 1992-1996 | 1.1947 | 1.0204 |
| 1997-2001 | 1.1655 | 1.0007 |
| 2002-2006 | 1 | 1 |
| 2007-2011 | 1.0052 | 0.9594 |
| 2012-2016 | 1.0499 | 0.9119 |
| 2017-2021 | 1.0886 | 0.9435 |
| Cohort |  |  |
| 1902 | 0.4891 | 0.9228 |
| 1907 | 0.5396 | 0.9521 |
| 1912 | 0.6719 | 1.0113 |
| 1917 | 0.7672 | 1.0433 |
| 1922 | 0.9023 | 1.0928 |
| 1927 | 1.0053 | 1.1156 |
| 1932 | 1.0349 | 1.1027 |
| 1937 | 1.0366 | 1.0985 |
| 1942 | 1.0542 | 1.1062 |
| 1947 | 1.0767 | 1.0957 |
| 1952 | 1.0532 | 1.0397 |
| 1957 | 1 | 1 |
| 1962 | 0.9731 | 0.9652 |
| 1967 | 0.9994 | 0.9352 |
| 1972 | 0.9763 | 0.911 |
| 1977 | 0.9314 | 0.8859 |
| 1982 | 0.8887 | 0.8661 |
| 1987 | 0.8806 | 0.8463 |
| 1992 | 0.8501 | 0.8653 |
| 1997 | 0.6907 | 0.8138 |
| 2002 | 0.4698 | 0.7745 |
| 2007 | 0.3613 | 0.7364 |
| 2012 | 0.3061 | 0.6637 |
| 2017 | 0.2915 | 0.5996 |
|  | P value# | |
|  | Mortality | Incidence |
| Net drift | <0.001 | <0.001 |
| All period rate ratios | <0.001 | <0.001 |
| All cohort rate ratios | <0.001 | <0.001 |
| All local drifts | <0.001 | <0.001 |
| # Wald Chi square tests were used for estimable functions | | |

**Supplementary Table 2.** The effects of age, period and cohort on the incidence and mortality rate of motor neuron diseases in Asia
